# Not just a covariate: circadian photoreception varies by age and sex

**DOI:** 10.1093/sleep/zsag095

**Published:** 2026-04-06

**Authors:** Delainey Wescott

**Affiliations:** Department of Psychiatry, University of Pittsburgh School of Medicine, Pittsburgh, PA, United States

Non-visual responses to environmental light influence human physiology and behavior and are an emerging marker of circadian health. Non-visual light is captured by specialized photoreceptors in the retina: intrinsically photosensitive retinal ganglion cells (ipRGCs). These ipRGCs contain the photopigment melanopsin, which responds to short-wavelength light [1]. Melanopic illuminance, or short-wavelength light that stimulates melanopsin, drives circadian photoreception [2]. ipRGCs project to the central circadian clock in the suprachiasmatic nucleus via the retinohypothalamic tract and have downstream effects on circadian entrainment, stimulation of clock gene expression, and pupil diameter [3–5]. The post illumination pupil response (PIPR) non-invasively indexes the neuronal firing response of ipRGCs by measuring sustained pupillary constriction following exposure to short-wavelength light [1].

Interindividual differences in light sensitivity can affect sleep and circadian health. In young adults, greater retinal light sensitivity has been linked with later sleep timing [6] while blunted light sensitivity has been associated with later sleep timing in adults with delayed sleep wake phase disorder [7]. In a community sample of adults, greater retinal light sensitivity was associated with later circadian phase assessed via dim light melatonin onset but only during the summer months when environmental light conditions are robust [8]. Sociodemographic features, including age and biological sex, may modulate these relationships. Women [9] and children [10] are more sensitive to light compared to men and adults when measuring circadian light sensitivity via melatonin suppression. Melatonin suppression may be driven by melanopsin [11], but it is unclear how retinal light sensitivity measures correspond to melatonin suppression [12]. Demographic comparisons of retinal light sensitivity methods have been largely underexplored.

van der Zwet et al. [13] examined how age and sex moderate the relationship between retinal light sensitivity and chronotype in a large sample of adults (*n* = 269) and children (*n* = 164) in a naturalistic setting. Retinal light sensitivity assessed with the PIPR did not differ by participant age, sex, or chronotype in adults or children. However, the authors showed compelling findings that the directionality of the relationship between retinal light sensitivity and chronotype varied by age and sex in adults. Greater light sensitivity was associated with later chronotype in younger adults, consistent with prior work noted above, although this relationship attenuated with age. Strikingly, in older adults the relationship reversed; blunted light sensitivity associated with later chronotype. These developmental findings are consistent with recent work linking blunted light sensitivity with delayed rest-activity acrophase in older adults [14]. Retinal physiology changes with age, including lens yellowing and decreasing pupil diameter [15]. Developmental changes in retinal physiology likely interact with behavioral patterns of light exposure to influence chronotype. van der Zwet et al. [13] further demonstrate the need to characterize circadian photoreception across the lifespan by biological sex. Sex differences in circadian photoreception may reflect differences in circadian period length [16], sensitivity to light [9] and/or light exposure behaviors [17]. Notably, hormonal fluctuations in menstrual phase, use of hormonal contraceptives, pregnancy, and menopause may impact light sensitivity, and possibly circadian photoreception, yet are rarely taken into account [18]. van der Zwet et al. [13] provide evidence that age and sex, at least in adulthood, contribute to substantial interindividual differences in circadian photoreception. Exploring age and sex effects directly, and not simply as covariates, is necessary to understand the complex interplay between physiology and behavior underlying circadian photoreception.

Interestingly, these relationships were not present in youth, ages 8–17. The authors acknowledged their youth sample was predominantly children with limited recruitment of adolescents, which may partly explain their null effects. Adolescence is a sensitive developmental period when the sleep and circadian system undergo marked changes, including potential changes in circadian photoreception. Children and adolescents differ in pupillary light responses [19] and light sensitivity varies from early to late adolescence [20]. It is possible that age and sex effects on circadian photoreception may not emerge until adolescence. Future work exploring circadian photoreception during the adolescent period may illuminate these developmental processes.

van der Zwet et al.’s [13] findings have important clinical implications for developing personalized light exposure recommendations and designing targeted light-based interventions. The combination of light exposure behaviors and light sensitivity influence sleep and circadian timing [21]. More recent modeling work suggests light-based interventions may be beneficial or maladaptive depending on the unique combination of an individual’s behavior and physiology [22]. Understanding how age and sex impact these relationships could allow for fine tuning model parameters to better characterize light’s effects on circadian health. As noted in van der Zwet et al. [13], this work is limited by not measuring habitual light exposure prior to pupillometry assessments. The authors took care to address this limitation by constricting data collection within two-weeks of the winter solstice ensuring participants were in a consistent photoperiod. Yet, two individuals living in identical photoperiods can exhibit marked differences in their light behaviors based on artificial light exposure, daylight accessibility, screen use, and sleep timing. Climate and geography can further shape behavioral patterns of light exposure [23] and extending this work across photoperiods and time zones is needed. Standardization of light reporting guidelines [24] and processing pipelines [25] will aid such efforts.

Finally, the unique study setting in van der Zwet et al. [13] should be lauded. By situating pupillometry assessments within a science museum, van der Zwet et al. [13] were able to investigate circadian photoreception in a real-world setting. Naturalistic environments may increase dissemination of sleep and circadian science and ideally promote uptake of community-based light-interventions. As the field moves towards personalized light exposure recommendations, we need creative and innovative study designs to understand how light impacts health for unique individuals in distinct environmental contexts. The findings from van der Zwet et al. [13] are an important contribution towards these aims.

## Disclosure statement

*Financial discloser:* The author has nothing to disclose.

*Non-financial disclosure:* The author serves on the Board of Directors for the Society for Light, Rhythms, and Circadian Health.
